# Serum Biomarkers of a Pro-Neuroinflammatory State May Define the Pre-Operative Risk for Postoperative Delirium in Spine Surgery

**DOI:** 10.3390/ijms241210335

**Published:** 2023-06-19

**Authors:** Johanna Ruhnau, Jonas Müller, Stephan Nowak, Sarah Strack, Denise Sperlich, Anna Pohl, Jasmin Dilz, Angelika Saar, Yannick Veser, Frederik Behr, Sebastian Rehberg, Taras Usichenko, Klaus Hahnenkamp, Johannes Ehler, Agnes Flöel, Henry W. S. Schroeder, Jan-Uwe Müller, Robert Fleischmann, Antje Vogelgesang

**Affiliations:** 1 Department of Neurology, University Medicine Greifswald, 17475 Greifswald, Germany; johanna.ruhnau@med.uni-greifswald.de (J.R.);; 2 Department of Neurosurgery, University Medicine Greifswald, 17475 Greifswald, Germany; 3 Department of Anesthesiology, Evangelisches Klinikum Bethel, 33617 Bielefeld, Germany; 4 Department of Anesthesiology, University Medicine Greifswald, 17475 Greifswald, Germany; 5 Department of Anesthesiology and Intensive Care Medicine, University Hospital Jena, 07743 Jena, Germany; 6 Center for Neurodegenerative Diseases Rostock/Greifswald, 18147 Rostock, Germany

**Keywords:** postoperative delirium, postoperative cognitive dysfunction, spine surgery, sTREM2, Gasdermin D, biomarker

## Abstract

Advances in spine surgery enable technically safe interventions in older patients with disabling spine disease, yet postoperative delirium (POD) poses a serious risk for postoperative recovery. This study investigates biomarkers of pro-neuroinflammatory states that may help objectively define the pre-operative risk for POD. This study enrolled patients aged ≥60 scheduled for elective spine surgery under general anesthesia. Biomarkers for a pro-neuroinflammatory state included S100 calcium-binding protein β (S100β), brain-derived neurotrophic factor (BDNF), Gasdermin D, and the soluble ectodomain of the triggering receptor expressed on myeloid cells 2 (sTREM2). Postoperative changes of Interleukin-6 (IL-6), Interleukin-1β (IL-1β), and C-reactive protein (CRP) were assessed as markers of systemic inflammation preoperatively, intraoperatively, and early postoperatively (up to 48 h). Patients with POD (*n* = 19, 75.7 ± 5.8 years) had higher pre-operative levels of sTREM2 (128.2 ± 69.4 pg/mL vs. 97.2 ± 52.0 pg/mL, *p* = 0.049) and Gasdermin D (2.9 ± 1.6 pg/mL vs. 2.1 ± 1.4 pg/mL, *p* = 0.29) than those without POD (*n* = 25, 75.6 ± 5.1 years). STREM2 was additionally a predictor for POD (OR = 1.01/(pg/mL) [1.00–1.03], *p* = 0.05), moderated by IL-6 (Wald-χ^2^ = 4.06, *p* = 0.04). Patients with POD additionally showed a significant increase in IL-6, IL-1β, and S100β levels on the first postoperative day. This study identified higher levels of sTREM2 and Gasdermin D as potential markers of a pro-neuroinflammatory state that predisposes to the development of POD. Future studies should confirm these results in a larger cohort and determine their potential as an objective biomarker to inform delirium prevention strategies.

## 1. Introduction

The proportion of people aged ≥65 increases at an unprecedented rate and is expected to reach about 60% in the year 2070 in the European Union [1]. Degenerative spine disease affects about 31–36% of these elderly people and accounts for about 10–14% of their disabilities [2].

Postoperative delirium (POD) is a serious perioperative neurocognitive disorder (pNCD) that affects about 25% of patients undergoing non-cardiac surgery and has a detrimental impact on functional recovery, complication rates, and healthcare costs [3]. Delayed neurocognitive recovery (dNCR) can follow POD or develop independently and evolve into postoperative cognitive dysfunction (POCD) in about 30–50% of patients, who endure neurocognitive symptoms for ≥3 months following surgery [4,5]. POCD is associated with the risk of long-term care dependency and institutionalization [6]. Symptoms persisting ≥12 months indicate a transition from postoperative to persistent neurocognitive disorders (NCD), including dementia [6,7].

Perioperative preventive interventions for POD in patients pre-operatively identified to be at risk, as well as early detection of POD in the postoperative period, are imperative means to prevent the development of long-term sequelae such as POCD and persistent NCD [7]. Unfortunately, biomarker profiles for an objective identification of patients at risk are unavailable in non-cardiac surgery [8]. Some candidate biomarkers, however, are available but require validation in spine surgery. A meta-analysis examining the relationship between inflammatory markers and POD and postoperative cognitive impairment indicated that patients who develop POD have significantly higher markers of systemic inflammation: For instance, preoperative Interleukin-6 (IL-6) and C-reactive protein (CRP) levels were higher than of those who did not develop POD. Furthermore, a large multicenter study of cerebrospinal fluid (CSF) in hip fracture demonstrates upregulation of metabolites from the kynurenine pathway in the serum that was tightly connected to metabolites in the CSF of patients with delirium [9].

With regard to the identification of pre-operative risk, markers of a pro-neuroinflammatory state might be particularly informative for the development of POD [10]. Microglial activity, a main contributor to neuroinflammation in neurodegeneration [11], is indicated in blood and CSF samples by the soluble ectodomain of the triggering receptor expressed on myeloid cells 2 (sTREM2) level [12]. A study by Henjum et al. demonstrated significantly elevated sTREM2 concentrations in CSF in patients with POD after hip fracture compared to non-delirious patients [12]. STREM2 is among others involved in the regulation of phagocytosis, clearance of apoptotic neurons, cytokine release, microglial proliferation and migration, and inhibition of proinflammatory responses in microglia [13,14]. Furthermore, S100 calcium-binding protein β (S100β) levels in blood samples tend to be higher postoperatively in POD patients and might aggravate cerebral damage by activating pro-inflammatory microglia [15,16]. In a study of patients aged ≥70 who underwent spine surgery and suffered POD, brain-derived neurotrophic factor (BDNF) decline was more pronounced during surgery (−74%) as compared to patients without POD (−50%) [17]. Gasdermin D is released after activation of the inflammasome by pathogen- and danger-associated molecular patterns (PAMPs and DAMPs) in the context of infection and/or injury [18,19]. It is also involved in the release of IL-1β but not in its synthesis [18,19,20]. In an experimental model, hippocampus-dependent memory deficits were accompanied by increased levels of Gasdermin D in the hippocampus [20]. To the best of our knowledge, the role of Gasdermin D in the POD has not yet been clarified.

Our study, “The cognitive dysfunction following elective spine surgery in elderly patients (CONFESS) study”, was designed as a prospective single-center observational study with the primary goal of determining the rate of POD after surgery [21]. Herein, we tested several sociodemographic, anesthesiological, and surgical characteristics. Although limiting the duration of surgery and meticulous management of intraoperative systolic blood pressure might decrease the risk of POD and POCD, we clearly found that spinal surgery per se performed with general anesthesia was not necessarily associated with POD [5]. Furthermore, the impact of medication on the incidence of POD was not significant. The current study was designed to assess the role of potential risk biomarkers for POD development using the data from our CONFESS study.

## 2. Results

### 2.1. Patient Population, Baseline Characteristics, Surgical Intervention, and Outcomes

Baseline-adjusted immediate (V1) and early (V2) postoperative serum levels of S100β, IL-6, IL-1β, Gasdermin D, sTREM2, BDNF, and CRP were examined in 19 patients suffering POD in comparison to 25 patients without POD. Both groups did not differ with respect to age and sex (75.7 ± 5.8 vs. 75.6 ± 5.1 years). Tabular overviews and comparisons of the baseline sociodemographic and surgical characteristics of these subpopulations are provided in Table 1. While demographic characteristics were comparable, the groups differed with respect to duration of surgery (with POD (min ± SD) vs. without POD: 256.3 ± 126.9 vs. 160.4 ± 80.5; *p* = 0.002) and extent of surgery (spinal levels with POD vs. without POD: 3.76 ± 1.75 vs. 2.1 ± 0.85; *p* < 0.001).

There were no significant pre-operative differences (V0) between markers of systemic inflammation between groups depending on their POD status following surgery. However, markers of a pro-neuroinflammatory state partially revealed significant differences. Serum concentrations of sTREM2 (128.18 ± 69.4 pg/mL, *p* = 0.049) and Gasdermin D (2.98 ± 1.64 pg/mL, *p* = 0.029) were significantly higher in patients that subsequently developed POD (Table 2). 

### 2.2. Changes in Biomarker Concentrations in the Post-Operative Period

Postoperative changes in markers of neuroinflammation did not significantly differ between patients with and without POD, except for increased S100β in the POD group as a potential indicator of astroglial damage on the first post-operative day (0.076 ± 0.073 g/mL vs. 0.027 ± 0.039 pg/mL, *p* = 0.003). There was a general increase in markers of systemic inflammation, which was most pronounced on the first postoperative day (V2.1, Table 3). Significant differences between markers of systemic inflammation were increased levels of IL-6 (mean difference: 37.55 ± 39.59 pg/mL) on the first and IL-1β (mean difference: 0.08 ± 0.18 pg/mL) and second (mean difference: 0.08 ± 0.16 pg/mL) postoperative days as compared to controls.

### 2.3. Preoperative Biomarkers for the Prediction of POD

**Figure 1:**
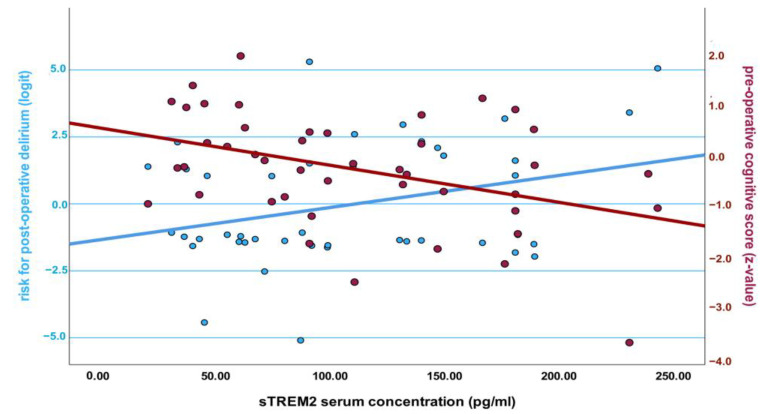
Illustration of the relationship between soluble ectodomain of triggering receptor expressed on myeloid cells 2 (sTREM2) baseline serum concentrations and the risk for postoperative delirium (POD) (left scale; β = 0.32, *p* = 0.03) and pre-operative cognitive abilities (right scale; β = −0.41, *p* < 0.01). The risk for POD is expressed as a logit resulting from a binary logistic regression including an adjustment for age, duration of surgery, and systemic IL-6 following surgery. Logit values need to be understood as the natural logarithm of the odds for POD and were chosen to enable presentation with cognitive scores in one figure. Negative logit values indicate a lower risk of POD, and vice versa. Cognitive scores are expressed as z-values, i.e., they are normalized to an age- and education-matched reference population.

We furthermore aimed to explore not only differences in biomarker concentrations but also their predictive value, which is important depending on well-established perioperative parameter risk factors such as age, duration of surgery, and post-operative systemic inflammation. The results are summarized in Table 4. Gasdermin D revealed only a marginal trend to predict POD (OR 1.50 [0.97–2.32], *p* = 0.07), which faded when adjusted for covariates in models 2 and 3. On the contrary, sTREM2 was marginally significant alone, but the predictive value of its pre-operative serum concentrations increased in adjusted models and turned out to be significant when postoperative systemic inflammation was considered. Since cognitive impairment shows a similar effect, i.e., increases the odds of POD depending on the duration of surgery and systemic inflammation, we were interested in whether sTREM2 might relate to inferior cognitive abilities. Indeed, there was a significant correlation between mean CERAD-NP scores and sTREM2 (Pearson correlation: −0.41, *p* = 0.006). This relationship is further illustrated in Figure 1. *p < 0.05.

## 3. Discussion

In the present study, we evaluated biomarkers of pro-neuroinflammatory states that may help objectively define the pre-operative risk for POD. We found that pre-operative levels of markers of a pro-neuroinflammatory state, i.e., sTREM2 and Gasdermin D, are associated with developing POD after spine surgery. While Gasdermin D seems to enhance the risk irrespective of systemic inflammation and the duration of surgery, the effects of sTREM2 were clearly related to the intervention. We furthermore found that elevated postoperative S100β, IL-6, and IL-1β concentrations are associated with an increased risk of developing delirium, and interventions reducing their perioperative release may decrease the risk for POD.

### 3.1. Markers of Systemic Inflammation and Risk for POD

Although the evidence on IL-6 and its association with POD is partially contested, our data suggest that higher IL-6 levels postoperatively, after adjusting for baseline concentration, are related to an increased risk of developing delirium. A recently published meta-analysis of 16 cohort research studies (a total of 2967 patients, 758 with POD and 2209 with non-POD) revealed that early after surgery, serum IL-6 levels were significantly higher in POD patients than in non-POD patients, suggesting that early serum inflammatory variables are likely to be predictors of POD [22]. Moreover, there is evidence that increased levels of IL-6 are related to a diminished cognitive recovery after delirium. In a mouse model of non-infectious acute lung injury, we have recently revealed that systemic IL-6 suppression reverses delirium-like neuronal alterations in the frontal cortex and hippocampus, indicating that IL-6 plays a central role in producing delirium-like structural phenotypes [23,24]. In addition, the treatment of the IL-6 receptor (IL-6R) blocking antibody tocilizumab prior to an open tibia fracture [25] reduced blood-brain barrier breakdown, enhanced hippocampus activation of microglia, and cognitive impairment. Unknown is whether cognitive deterioration could be mediated by IL-6-induced delirium and whether IL-6 inhibition could promote recovery after POD. Growing evidence suggests that POD is associated with higher levels of inflammatory cytokines, such as IL-6 and CRP [26,27]. Nevertheless, the findings of studies are conflicting, with some reporting higher plasma IL-6 levels in individuals with delirium [28,29] and others detecting different results [30]. These differences in the results may be due to the types of surgery and different methods of analyzing inflammatory markers. To our knowledge, our study is the first in spinal surgery to detect plasma IL-6 values as a possible marker to evaluate POD outcomes as part of a multifactorial approach. Van Munster et al. were able to demonstrate that the S100β concentration is elevated in 81% of POD [15] patients with hip fractures and surgical therapy. Similarly, Rasmussen et al. in patients (mean age 68) after abdominal surgery [31] and Liu et al. in a meta-analysis found a significant rise in S100β postoperatively, which is related to an elevated POD risk [32]. Hence, our findings that a higher postoperative S100β level predicts POD are consistent with previous findings and suggest a crucial role for astrocyte activation, malfunction, and blood-brain barrier permeability in the development of POD. In conjunction with Khan et al.‘s observation that normal S100β levels on day 8 correspond with delirium resolution, this suggests that activated astrocytes play a role in the maintenance of delirium [33]. Furthermore, S100β is known to generate reactive oxygen species, which are involved in oxidative stress and long-term neurodegeneration [34]. Thus, higher postoperative serum biomarkers like S100β or IL-6 could represent a mirror of the inflammatory processes in the CNS during delirium.

### 3.2. Markers of Neuro-Inflammation and Risk for POD

Gasdermin D is particularly released in response to infection and/or injury, as well as an imbalance of homeostasis in the CNS [19,20]. Since our data demonstrate that the higher the concentration before surgery, the more likely it is that POD will develop, it seems that people who develop POD are already in a proinflammatory state. This seems to predispose patients to develop POD and is in line with the neuroinflammation hypothesis [18]. Moreover, Gasdermin D is increased in neurodegenerative processes in the hippocampus, which has already been demonstrated in animal experiments [20].

In the CONFESS study, only subjects > 60 years of age were recruited, thus providing an age range for which it is reasonable to presume that neurodegenerative processes have already begun and are continuing. The hippocampus is primarily responsible for memory function, neurogenesis, and affect control. Increased Gasdermin D thereby triggered increased pryoptosis, IL-1β release, and increased inflammasome expression in the hippocampus, leading to impairment of these functions, which fits the symptomatology of POD and also POCD [18,19]. 

Excessive neuroinflammation leads to an imbalance in the central nervous system due to an overabundance of proinflammatory factors and a deficiency of anti-inflammatory factors. This can result in abnormalities in executive functions and working memory similar to those observed in postoperative delirium. The excessive cytokine concentration simultaneously hinders astrocyte activation, which limits glutamate-dependent calcium influx into astrocytes and culminates in irreversible synaptic damage. Concurrently, the production of cytokines by astrocytes, such as Monocyte Chemoattractant Protein-1 (MCP-1), causes significant microglial activation [35]. This interaction between astrocytes and microglia exacerbates the systemic inflammatory response [36]. Moreover, when microglia age, they become less sensitive to anti-inflammatory mediators, which promotes excessive inflammation and makes it difficult to establish homeostasis [37]. 

In 2020, Halaas et al. demonstrated that in cognitively unrestricted subjects ≥ 65 years of age (delirium as an exclusion criterion), elevated baseline sTREM2 in CSF is associated with accelerated cortex thinning in the left hemisphere temporal cortex and bilateral hippocampal atrophy [38]. Preoperatively elevated sTREM2 as a predictor of POD can be taken as an indication for the microglia priming theory [13,14]. Even before surgery, there appears to be an enhanced microglial activity status in POD patients (as measured by sTREM2 concentration), which is both pro-inflammatory (mainly IL-1β release is boosted) and functions as a CNS amplifier of systemic inflammation [13]. In the context of POD, sTREM2 has been detected preoperatively in subjects with early dementia. For this purpose, Henjum et al. studied patients with hip fractures and their surgical treatment [12]. Only recently, Wang et al. revealed that a higher value of postoperative sTREM2 in the CSF increased the risk of early POCD. These data were adjusted for POD duration [39]. It is likely that sTREM2-mediated microglial activation induces acute neuroinflammation. A solid relationship between neuroinflammation and POCD has been reported in orthopedic patients, which fully demonstrates the critical role of neuroinflammation in POCD development [40]. Microglial activation and subsequent neuroinflammatory response frequently accompany the early progress of amyloid beta accumulation, tau pathology, cerebrovascular injury, and cognitive impairment [41]. The preoperative activation of the central nervous system by sTREM2 and Gasdermin D could influence not only short-term consequences like the risk of developing a POD but also longer-term complications, i.e., POCD, by creating a neuro-inflammatory milieu.

## 4. Limitations

In the main trial, CONFESS, we intended to test the major hypothesis on 182 patients; however, this observational trial had to be terminated owing to the COVID pandemic, which would have resulted in a selection bias due to the triage of non-emergent procedures. Nonetheless, our primary hypothesis that the duration of surgery influences POD was confirmed given the larger than expected effect size. For the analysis of biomarkers, we matched patients with and without POD by age, sex, and pre-operative physical fitness (using the American Society of Anesthesiologists physical status classification system). Analyses of biomarkers were defined as a secondary endpoint and analyzed exploratively, which decreased the evidence provided by our findings. Nonetheless, investigations were conducted as prospectively registered and planned in the study protocol, which should diminish type 1 error. Since effect sizes are now available, future studies can be adequately powered to investigate their impact as a primary or predefined secondary endpoint with sufficient power. Differences in numbers in both groups are due to a lack of blood material in the delirium group. The availability of blood samples was only identified after having matched POD cases with controls, rendering the exclusion of already selected controls a potential source of bias. We hence decided to keep the group sizes as they turned out to be without further inference with sample selection to avoid selection bias. 

Although repeated CSF sampling would have been desirable to analyze and compare levels of biomarkers in serum to the CNS environment, such a study design would create significant ethical and practical challenges. It could be suggested that in patients with preoperatively elevated biomarkers, POD should be explicitly pointed out again in the context of the surgical/anesthetic information. After surgery, these patients could be monitored more closely regarding POD and especially POCD. If necessary, cerebral imaging should also be discussed with these patients to determine whether they have suffered structural brain damage perioperatively. Spinal surgery per se was not necessarily associated with POCD in the CONFESS study if these have a functional gain (i.e., postoperative Barthel index increases) and do not suffer POD [5]. Nonetheless, it is possible that the preclinical state of dementia/or neurodegenerative diseases, which induces a proinflammatory state of biomarkers, might be responsible for the higher risk of POD in the delirium cohort.

## 5. Materials and Methods

### 5.1. Study Registration and Data Availability

The cognitive dysfunction following elective spine surgery in elderly patients (CONFESS) study is a prospective single-center observational study. The trial was approved by the Institutional Review Board of the University of Greifswald (BB 192/17) and prospectively registered (clinicaltrials.gov, NCT03486288) [21].

### 5.2. Setting and Participants

The study was jointly conducted by the Departments of Neurosurgery and Neurology in cooperation with the Department of Anesthesiology at a 950-bed tertiary care university hospital. Patient recruitment started in February 2018 and was stopped in March 2020 due to the triage of non-emergent surgical procedures during the SARS-CoV-2 pandemic [42]. The primary analysis of this cohort was whether the duration of surgery influences POD. The sample size simulation yielded a sample size of *n* = 182. Due to the SARS-CoV-2 pandemic, we did not reach this planned sample size. Nonetheless, our primary hypothesis was confirmed given the larger than expected effect size [5]. Data collection was completed in March 2021. Patients aged ≥60 years presenting to the outpatient clinic of the Department of Neurosurgery were eligible. Inclusion criteria were elective spine surgery without opening the dura, the ability to provide informed consent himself/herself, and being a native German speaker. Exclusion criteria were any diagnosis of neurodegenerative/psychiatric disease, central nervous system (CNS)-active medication (e.g., antidepressants, antipsychotics, sedatives), inability to participate in follow-up, participation in an interventional trial, electronic or displaceable metallic implants, or active neoplasms.

### 5.3. Study Design and Delirium Assessment

A tabular overview of the visit plan and endpoints is given in Table 5. The postoperative period (V2) consisted of delirium assessments three times daily for at least 72 h using an established screening instrument (Nursing Delirium Screening Scale, Nu-DESC; cut-off ≥ 2 points) [43]. The diagnosis of POD had to be confirmed using DSM-5 criteria. Since POD is not related to secondary complications (e.g., infection), it is expected to develop within 72 h [44]. Biosamples were only investigated on V0, V1, and on two days in the postoperative period (V2.1 and V2.2). For biomarkers, we analyzed the sera of 44 patients out of the total cohort of 99 patients enrolled in the CONFESS study. Patients who suffered POD and patients who did not develop delirium were matched for age, sex, and pre-operative physical fitness (using the American Society of Anesthesiologists physical status classification system).

### 5.4. Assessment of Secondary Outcome Parameters

Sociodemographic characteristics (age, gender, education, graduation, and work status), substance abuse (alcohol and nicotine), medication, and medical history were recorded as suggested [45]. Cognitive abilities were evaluated at V0 using the “Consortium to Establish a Registry for Alzheimer’s Disease Plus test battery” (CERAD-NP) [46]. The CERAD-NP includes assessments of orientation, visual naming, phonematic speed, semantic fluency, verbal episodic memory (encoding, error control, recall, and discriminability), non-verbal episodic memory (encoding, recall), visuoconstruction abilities, attention, executive speed, and functions. In this study, the total score (i.e., the mean of the z-values of all domains) was used to assess global pre-operative cognitive performance [47].

### 5.5. Perioperative Surgical and Anesthesiological Procedures

Surgical and anesthetic procedures adhered to national clinical guidelines and in-house standard operating procedures. These were not adapted for study purposes. Spine surgery was performed by experienced board-certified neurosurgeons. Patients were generally operated on in a prone position except for ventral fusion (supine) without compression of the abdomen by using proper positioning cushions and covered with a thermal blanket. Assistive devices included an operating microscope and a mobile X-ray device. Procedures were categorized by the number of spine levels, perceived level of complexity (simple, intermediate, or complex), and type of intervention (ventral or dorsal cervical decompression and/or fusion; thoracic and/or lumbar multilevel decompression and/or fusion; kyphoplasty).

Anesthesia started with oral premedication with midazolam (0.1 mg·kg^−1^), if preoperative excitement was present, and was induced by intravenous injection of sufentanil (0.3–0.6 mg·kg^−1^) and propofol (1.5–2.5 mg·kg^−1^). Muscular relaxation was performed by intravenous injection of cisatracurium (1.5 mg·kg^−1^). Anesthesia was maintained with sevoflurane at a target alveolar concentration of 0.8–1.0 × MAC. Adequate anesthetic depth was verified via continuous monitoring of the bispectral index and real-time electroencephalography waveforms along the scalp. Estimated insensitive fluid losses were replaced by an intravenous infusion of an isotonic blood electrolyte solution without lactate. Continuous recording of vital parameters started about thirty minutes before induction of anesthesia and included five lead electrocardiography, pulse oximetry (SpO_2_), and non-invasive blood pressure (BP) measurement (five-minute interval) or through an arterial catheter if required. Hypotensive situations were managed through fluid challenges and continuous medication with norepinephrine at the discretion of the anesthesiologist in charge.

### 5.6. Measurement of Biomarkers

The sera for the analysis of biomarkers were sampled on V0–V2 and stored at −80 °C until analysis. The enzyme-linked immunosorbent assays (ELISA) were performed following the sandwich ELISA principle using the ELISA reader Infinite M200 Pro, TECAN Group Ltd., Männedorf, Switzerland. The corresponding manufacturer’s instructions were followed when performing the ELISAs (Human IL-6 Uncoated ELISA; Human TREM2 ELISA Kit; Human BDNF ELISA Kit; Invitrogen, ThermoFisher Scientific, Waltham, MA, USA; Gasdermin D ELISA, Horizon Bioscience Discovery Ltd., Cambridge, UK). However, deviating from this, the standard curve of the IL-1β-ELISA (Interleukin-1beta high sensitivity ELISA; IBL International GmbH, Hamburg, Germany,) was extended by a lower concentration by adding a further dilution step (1:2). The subsequent photometric measurement of the absorbance was performed at a wavelength of 450 nm by the appropriate microplate reader (ELISA reader: Multiskan EX, Thermo Electron Corporation, Waltham, MA, USA). The generation of the standard curve and calculation of the concentrations are performed with the statistical software GraphPad PRISM 8.0.1.

Values for CRP (all measured with the Dimension Vista, Siemens Healthcare Diagnostics, Eschborn, Germany) were measured in the central laboratory facility of the University Medicine Greifswald. 

### 5.7. Data Management, Sample Size Considerations, and Statistics

Study data was transferred from paper to an electronic case report form (CentraXX, Kairos GmbH, Bochum, Germany) that included real-time verification of completeness, range, and type of data entered. Statistical evaluations were performed using SPSS (v28, IBM, Armonk, NY, USA). Descriptive statistics are either reported as frequencies or group means ± standard deviation. Results from inferential statistics are reported with the level of significance (*p*-value) and, if applicable, coefficients (i.e., odds ratios (OR) or unstandardized beta coefficients with their 95% confidence interval (95% CI) in logistic or linear regression models, respectively).

All datasets were tested for adherence to the Gaussian distribution by Shapiro-Wilk tests and/or distribution in Q–Q plots. Analyses of biomarker profiles were considered exploratory. This being said, group-wise comparisons (POD vs. non-POD) of biomarker concentrations before surgery (i.e., potential predictors) and following surgery (i.e., pathomechanisms related to the development of POD) were conducted using unpaired *t*-tests. Significant values from these exploratory analyses were then included in hypothesis-driven predictive multivariable logistic regression models, including the POD status as a binary dependent variable. Significant predictors were then individually assessed in a univariate analysis, including only the potentially predictive biomarker (model 1), then duration of surgery and age as well-established covariates (model 2), and finally markers of post-operative systemic inflammation possibly affecting a pro-neuroinflammatory pre-operative state (model 3). Pearson’s analyses were performed based on adherence to the Gaussian distribution. Correlation analyses were conducted to explore the relationship between risk biomarkers and pre-operative cognitive abilities.

### 5.8. Writing

In part, QuillBot (https://quillbot.com/) was used for grammar improvement and spell checking.

## 6. Conclusions and Outlook

Serum biomarkers associated with presumed pathophysiological mechanisms underlying the development of or risk for delirium may help identify patients at risk and inform clinical decision-making, i.e., adjust modifiable risk factors to individual risk profiles. This being said, adjusting the duration of surgery provides a readily available means to reduce the risk of POD. Our study revealed that preoperative levels of markers of a pro-neuroinflammatory state, i.e., sTREM2 and Gasdermin D, are associated with POD development. Furthermore, sTREM2 and IL-6, as modulators of microglia proliferation, seem to be involved in POD pathophysiology and increase the odds of POD. Future studies should confirm these results in a larger cohort and determine their potential as an objective means to inform delirium prevention strategies. Especially whether all observed markers might be able to predict POD even better than preoperative cognitive testing as the gold standard has to be evaluated in the future. 

## Figures and Tables

**Table 1:** Summary of the sociodemographic characteristics and medical history of the study population. Values are given either as frequencies or group mean ± standard deviation. n.s. = non-significant difference. “Consortium to Establish a Registry for Alzheimer’s Disease Plus test battery” (CERAD): Differences in pre-operative biomarker concentrations.

	Delirium Group	Non Delirium Group	*p*-Value of Difference
Number of patients	19	25	
Gender	male = 8 (42.1%)	male = 12 (50%)	n.s.
	female = 11 (57.9%)	female = 12 (50%)	n.s.
Age in years	75.7 ± 5.8	75.6 ± 5.1	n.s.
Years of education	8.63 ± 1.42	9.5 ± 1.69	n.s.
Duration of POD (days)	1.6 ± 1.5	-	n.s.
Preoperative cognition (CERAD)	−0.9 ± 1.1	0.0692 ± 0.8	<0.001
Duration of surgery	256.3 ± 126.9	160.4 ± 80.5	0.002
Spinal levels	3.76 ± 1.75	2.1 ± 0.85	<0.001

**Table 2:** Pre-operative differences in biomarker (V0) concentrations. All values are given as means ± standard deviation (Std. Deviation). *p*-values result from unadjusted unpaired *t*-tests between groups; * significant differences are indicated by bold font (*p* < 0.05). sTREM2 (soluble ectodomain of triggering receptor expressed on myeloid cells 2); brain-derived neurotrophic factor (BDNF); Interleukin-6 (IL-6); Interleukin-1beta (IL-1β); C-reactive protein (CRP); S100 calcium-binding protein β (S100β); postoperative delirium (POD).

Biomarker	POD Status	Number of Patients	Mean	Std. Deviation	*p*-Value
sTREM2	No	25	97.24	52.03	0.049 *
	Yes	19	128.18	69.42	
Gasdermin D	No	25	2.09	1.38	0.029 *
	Yes	19	2.98	1.64	
S100β	No	25	0.06	0.04	0.185
	Yes	19	0.07	0.03	
BDNF	No	25	188.93	77.15	0.387
	Yes	19	182.27	72.83	
IL-6	No	25	8.73	33.00	0.443
	Yes	19	10.23	35.11	
IL-1β	No	25	0.01	0.03	0.324
	Yes	19	0.01	0.03	
CRP	No	25	11.77	23.65	0.494
	Yes	19	11.86	11.27	

**Table 3:** Post-operative differences in biomarker concentrations. All values are given as means ± standard deviation (Std. Deviation). Significant differences between groups based on unadjusted unpaired *t*-tests between groups are indicated by bold font (*p* < 0.05). Baseline-adjusted values are shown as ΔV1; ΔV2.1; ΔV2.2.

			ΔV1		ΔV2.1		ΔV2.2	
	POD Status	N	Mean	Std. Deviation	Mean	Std. Deviation	Mean	Std. Deviation
sTREM2	No	25	−11.86	35.02	−37.27	103.32	−32.72	102.82
	Yes	19	−28.58	53.04	44.86	324.53	48.97	315.12
Gasdermin D	No	25	−0.73	1.13	−0.26	0.75	−0.34	1.37
	Yes	19	−0.99	1.40	−0.31	1.27	−0.07	0.91
S100β	No	25	0.65	0.72	**0.03**	**0.04**	0.03	0.05
	Yes	19	0.69	0.58	**0.08**	**0.07**	0.07	0.12
ΔBDNF	No	25	−43.61	56.77	−60.45	50.92	−70.00	51.02
	Yes	19	−47.39	39.36	−66.23	57.08	−58.78	54.09
IL-6	No	25	7.11	18.06	**14.95**	**20.66**	21.30	33.92
	Yes	19	17.66	34.30	**37.55**	**39.59**	33.36	40.62
IL-1β	No	25	0.00	0.06	**0.01**	**0.03**	**0.01**	**0.05**
	Yes	19	0.03	0.05	**0.08**	**0.18**	**0.08**	**0.16**
CRP	No	25	−0.81	3.39	30.28	28.35	93.41	66.79
	Yes	19	−3.36	7.01	39.45	33.23	108.89	61.21

V1: perioperative; V2.1: first postoperative day; V2.2.: second postoperative day; sTREM2 (soluble ectodomain of triggering receptor expressed on myeloid cells 2); brain-derived neurotrophic factor (BDNF); Interleukin-6 (IL-6); Interleukin-1beta (IL-1β); C-reactive protein (CRP); S100 calcium-binding protein β (S100β); postoperative delirium (POD).

**Table 4:** Post-operative differences in biomarker concentrations. All values are given as means ± standard deviation. Significant differences between groups are based on unadjusted unpaired *t*-tests between groups. Interleukin-6 (IL-6); sTREM2 (soluble ectodomain of triggering receptor expressed on myeloid cells 2).

		Model 1		Model 2		Model 3	
		Odds Ratio	*p*-Value	Odds Ratio	*p*-Value	Odds Ratio	*p*-Value
sTREM2	sTREM2	1.01 [1.00–1.02]	0.10	1.01 [1.00–1.02]	0.09	1.01 [1.00–1.03]	0.05
	Age	…	…	1.03 [0.89–1.18]	0.74	1.05 [0.90–1.21]	0.56
	Duration	…	…	1.11 [1.03–1.20]	0.01	1.10 [1.01–1.20]	0.02
	IL-6	…	…	…	…	1.03 [1.00–1.06]	0.07
Gasdermin D	Gasdermin D	1.50 [0.97–2.32]	0.07	1.25 [0.77–2.03]	0.37	1.17 [0.72–1.89]	0.53
	Age	…	…	1.04 [0.89–1.19]	0.64	1.06 [0.91–1.24]	0.47
	Duration	…	…	1.09 [1.01–1.18]	0.03	1.08 [1.00–1.17]	0.05
	IL-6	…	…	…	…	1.02 [1.00–1.05]	0.15

**Table 5:** Summary of the recruitment process and visit plan. Patients were investigated on V0 (pre-operative state), V1 (perioperative), and on two days in the postoperative period (V2.1 and V2.2). V2 included compulsory examinations for the first three days, which were discontinued if the patients did not develop POD [44]. × depicts timepoint of assessments.

		Study Period			
		Preoperative	Perioperative	Postoperative	
	Enrolment	V0	V1	V2.1	V2.2
Timepoint		−7 d ± 7	0	1 d	2 d
Eligibility screen	×				
Informed consent	×				
Demographic data		×			
Medical history		×			
Cognitive testing		×			
Delirium screening		×	×	3×	3×
Blood samples		×	×	×	×

## Data Availability

Data are available from the corresponding author upon reasonable request. Unrestricted publication of datasets is not covered by local directives of the General Data Protection Regulation (EU) 2016/679.
